# Evaluation of a pharmacogenetic test in Thailand for abacavir hypersensitivity screening in human immunodeficiency virus infection

**DOI:** 10.1186/2045-7022-4-S3-P122

**Published:** 2014-07-18

**Authors:** Sukasem Chonlaphat, Chonlaphat SUKASEM, Napatrupron Koomdee, Siwalee Santon, Montri Chamnanphon, Thawinee Jantararoungtong

**Affiliations:** 1 Ranathibodi Hospital Mahidol University, Thailand; 2 Laboratory for Pharmacogenomics, Faculty of Medicine Ramathibodi Hospital, Mahidol University, Bang, Department of Pathology, Thailand

## 

Abacavir hypersensitivity reaction (ABC-HSR) is associated with the presence of HLA-B*5701 allele. Alternative tests for ABC-HSR associated allele determination are needed where sequence-based HLA typing is not available, particularly in resource-limited settings or developing countries. This study focused on the development and evaluation of two HLA-B*5701 tagging SNPs (single nucleotide polymorphism) HCP5 (HLA complex P5) rs2395029 and TNF (tumor necrosis factor) rs3093726 genotyping assays. Two hundred and thirteen purified genomic DNA samples were used to evaluate the performance characteristics of a HLA-B*5701 screening method based on allele-specific polymerase chain reaction (AS-PCR) with melting curve analysis. HCP5 rs2395029 and TNF rs3093726 were also genotyped using simple probe real-time PCR assay. All samples were successfully genotyped wherein AS-PCR genotyping provided 100% sensitivity, specificity, positive predictive value (PPV) and negative predictive value (NPV) when compared with specific HLA-B status by sequencing based assay. When comparing the AS-PCR screening method with the HCP5 rs2395029 and TNF rs3093726 genotyping method, the former had 100% sensitivity, 100% specificity, 100% PPV and 100% NPV using a simple probe; while the latter one had 95.24% sensitivity, 100% specificity, 100% PPV and 99.48% NPV, respectively. In conclusion, our study lends support on a molecular tool for pharmacogenetic screening in resource-limited settings. Thus, serious drug hypersensitivity associated with ABC may potentially be reduced in Thailand by further evaluation of the proposed assay in clinical practice.

